# 2-[(3-Propyl­sulfanyl-5-*p*-tolyl-4*H*-1,2,4-triazol-4-yl)imino­meth­yl]phenol

**DOI:** 10.1107/S1600536811029849

**Published:** 2011-08-02

**Authors:** Wei Wang, Qing-lei Liu, Chao Xu, Wen-peng Wu, Yan Gao

**Affiliations:** a School of Perfume and Aroma Technology, Shanghai Institute of Technology, Shanghai 200235, People’s Republic of China; b School of Chemical Engineering, University of Science and Technology LiaoNing, Anshan 114051, People’s Republic of China

## Abstract

In the title mol­ecule, C_19_H_20_N_4_OS, the two benzene rings form dihedral angles of 16.2 (1) and 12.0 (1)°, respectively, with the central triazole ring. In the crystal, inter­molecular O—H⋯N hydrogen bonds link mol­ecules into chains in the [010] direction.

## Related literature

For standard values of the bond lengths, see: Allen *et al.* (1987 ▶). For the crystal structure of a related compound, see: Wang *et al.* (2011 ▶).
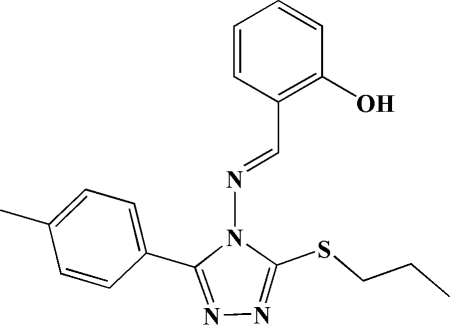


## Experimental

### 

#### Crystal data

C_19_H_20_N_4_OS
*M* *_r_* = 352.45
Monoclinic, 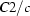

*a* = 22.682 (2) Å
*b* = 18.1736 (15) Å
*c* = 9.1557 (8) Å
β = 109.678 (7)°
*V* = 3553.7 (5) Å^3^
*Z* = 8
Mo *K*α radiation
μ = 0.20 mm^−1^
*T* = 113 K
0.20 × 0.16 × 0.12 mm

#### Data collection

Rigaku Saturn CCD area-detector diffractometer
Absorption correction: multi-scan (*CrystalClear*; Rigaku/MSC, 2005 ▶) *T*_min_ = 0.962, *T*_max_ = 0.977
16201 measured reflections
3499 independent reflections
3206 reflections with *I* > 2σ(*I*)
*R*_int_ = 0.061

#### Refinement

*R*[*F* ^2^ > 2σ(*F* ^2^)] = 0.059
*wR*(*F* ^2^) = 0.122
*S* = 1.14
3499 reflections
232 parameters
H atoms treated by a mixture of independent and constrained refinement
Δρ_max_ = 0.28 e Å^−3^
Δρ_min_ = −0.24 e Å^−3^

### 

Data collection: *CrystalClear* (Rigaku/MSC, 2005 ▶); cell refinement: *CrystalClear*; data reduction: *CrystalClear*; program(s) used to solve structure: *SHELXS97* (Sheldrick, 2008 ▶); program(s) used to refine structure: *SHELXL97* (Sheldrick, 2008 ▶); molecular graphics: *SHELXTL* (Sheldrick, 2008 ▶); software used to prepare material for publication: *SHELXTL*.

## Supplementary Material

Crystal structure: contains datablock(s) global, I. DOI: 10.1107/S1600536811029849/cv5131sup1.cif

Structure factors: contains datablock(s) I. DOI: 10.1107/S1600536811029849/cv5131Isup2.hkl

Supplementary material file. DOI: 10.1107/S1600536811029849/cv5131Isup3.cml

Additional supplementary materials:  crystallographic information; 3D view; checkCIF report

## Figures and Tables

**Table 1:** Hydrogen-bond geometry (Å, °)

*D*—H⋯*A*	*D*—H	H⋯*A*	*D*⋯*A*	*D*—H⋯*A*
O1—H1⋯N2^i^	0.95 (3)	1.71 (3)	2.658 (2)	175 (3)

Symmetry code: (i) 
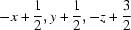
.

**Fig. 1.**
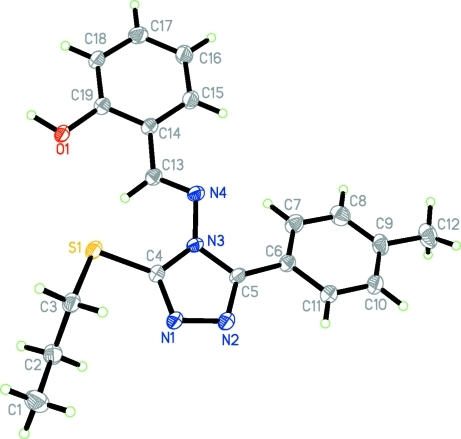
View of the molecule of (I) showing the atom-labelling scheme. Displacement ellipsoids are drawn at the 50% probability level.

## supplementary crystallographic information

### Comment

In continuation of structural study of Mannich bases derivatives synthesized by reactions of the amino heterocycles and aromatic aldehydes in our group (Wang *et al.*, 2011), we present here the crystal structure of the title compound, (I).

In (I) (Fig.1), all the bond lengths and angles are normal (Allen *et al.*, 1987). The C5 atom in the triazole ring deviates form the normal C*_sp_*^2^ hybridization state having the bond angles of 108.4 (2)° (N2—C5—N3) and 127.6 (2)° (N3—C5—C6), respectively. Rings C6—C11 and C14—C19 are inclined with repect to the 1,2,4-triazole ring at 16.2 (2)° and 12.0 (2)°, respectively. Two benzene rings form a dihedral angle of 18.0 (2)°.

In the crystal structure, intermolecular O—H···N hydrogen bonds (Table 1) link the adjacent molecules into chains in [010].

### Experimental

The title compound was synthesized by the reaction of salicylic aldehyde (2.0 mmol) and 4-amino-3-propylthio-5-*p*-tolyl-1,2,4-triazole (2.0 mmol) by refluxing in ethanol. The reaction progress was monitored *via* TLC. The resulting precipitate was filtered off, washed with cold ethanol, dried and purified to give the target product as colourless solid in 89% yield. Crystals of (I) suitable for single-crystal X-ray analysis were grown by slow evaporation of a solution in chloroform-ethanol (1:1).

### Refinement

The H atom attached to O atom was located in a difference map and refined isotropically. C-bound H atoms were positioned geometrically  (C—H = 0.95–0.99 Å) and refined as riding, with *U*_iso_(H) = 1.2-1.5 *U*_eq_(C).

### Crystal data

**Table:**

C_19_H_20_N_4_OS	*F*(000) = 1488
*M**_r_* = 352.45	*D*_x_ = 1.318 Mg m^−^^3^
Monoclinic, *C*2/*c*	Mo *K*α radiation, λ = 0.71073 Å
Hall symbol: -C 2yc	Cell parameters from 5676 reflections
*a* = 22.682 (2) Å	θ = 1.9–27.9°
*b* = 18.1736 (15) Å	µ = 0.20 mm^−^^1^
*c* = 9.1557 (8) Å	*T* = 113 K
β = 109.678 (7)°	Prism, colourless
*V* = 3553.7 (5) Å^3^	0.20 × 0.16 × 0.12 mm
*Z* = 8	

### Data collection

**Table:**

Rigaku Saturn CCD area-detector diffractometer	3499 independent reflections
Radiation source: rotating anode	3206 reflections with *I* > 2σ(*I*)
multilayer	*R*_int_ = 0.061
Detector resolution: 14.22 pixels mm^-1^	θ_max_ = 26.0°, θ_min_ = 1.9°
φ and ω scans	*h* = −27→27
Absorption correction: multi-scan (*CrystalClear*; Rigaku/MSC, 2005)	*k* = −21→22
*T*_min_ = 0.962, *T*_max_ = 0.977	*l* = −11→11
16201 measured reflections	

### Refinement

**Table:**

Refinement on *F*^2^	Primary atom site location: structure-invariant direct methods
Least-squares matrix: full	Secondary atom site location: difference Fourier map
*R*[*F*^2^ > 2σ(*F*^2^)] = 0.059	Hydrogen site location: inferred from neighbouring sites
*wR*(*F*^2^) = 0.122	H atoms treated by a mixture of independent and constrained refinement
*S* = 1.14	*w* = 1/[σ^2^(*F*_o_^2^) + (0.0382*P*)^2^ + 4.5494*P*] where *P* = (*F*_o_^2^ + 2*F*_c_^2^)/3
3499 reflections	(Δ/σ)_max_ = 0.001
232 parameters	Δρ_max_ = 0.28 e Å^−^^3^
0 restraints	Δρ_min_ = −0.24 e Å^−^^3^

### Special details

**Table:**

Geometry. All e.s.d.'s (except the e.s.d. in the dihedral angle between two l.s. planes) are estimated using the full covariance matrix. The cell e.s.d.'s are taken into account individually in the estimation of e.s.d.'s in distances, angles and torsion angles; correlations between e.s.d.'s in cell parameters are only used when they are defined by crystal symmetry. An approximate (isotropic) treatment of cell e.s.d.'s is used for estimating e.s.d.'s involving l.s. planes.
Refinement. Refinement of *F*^2^ against ALL reflections. The weighted *R*-factor *wR* and goodness of fit *S* are based on *F*^2^, conventional *R*-factors *R* are based on *F*, with *F* set to zero for negative *F*^2^. The threshold expression of *F*^2^ > σ(*F*^2^) is used only for calculating *R*-factors(gt) *etc*. and is not relevant to the choice of reflections for refinement. *R*-factors based on *F*^2^ are statistically about twice as large as those based on *F*, and *R*- factors based on ALL data will be even larger.

### Fractional atomic coordinates and isotropic or equivalent isotropic displacement parameters (Å^2^)

**Table:**

	*x*	*y*	*z*	*U*_iso_*/*U*_eq_	
S1	0.28535 (3)	0.18666 (3)	0.66027 (7)	0.02339 (18)	
O1	0.22719 (7)	0.34890 (9)	0.84176 (19)	0.0231 (4)	
H1	0.2327 (15)	0.3982 (19)	0.816 (4)	0.065 (11)*	
N1	0.28721 (9)	0.03831 (11)	0.6965 (2)	0.0231 (5)	
N2	0.25373 (9)	−0.01562 (11)	0.7421 (2)	0.0234 (5)	
N3	0.21532 (9)	0.09105 (10)	0.7742 (2)	0.0190 (4)	
N4	0.17143 (9)	0.14097 (10)	0.7951 (2)	0.0206 (4)	
C1	0.43083 (12)	0.09850 (16)	0.5327 (3)	0.0357 (7)	
H1A	0.4081	0.0552	0.4785	0.053*	
H1B	0.4737	0.0844	0.5946	0.053*	
H1C	0.4320	0.1359	0.4566	0.053*	
C2	0.39763 (11)	0.12982 (14)	0.6392 (3)	0.0257 (6)	
H2A	0.3961	0.0919	0.7156	0.031*	
H2B	0.4215	0.1725	0.6969	0.031*	
C3	0.33133 (11)	0.15388 (13)	0.5455 (3)	0.0240 (5)	
H3A	0.3092	0.1118	0.4819	0.029*	
H3B	0.3337	0.1935	0.4735	0.029*	
C4	0.26334 (10)	0.10185 (12)	0.7148 (3)	0.0194 (5)	
C5	0.21029 (11)	0.01597 (12)	0.7866 (3)	0.0196 (5)	
C6	0.16446 (11)	−0.02466 (13)	0.8370 (3)	0.0227 (5)	
C7	0.12894 (12)	0.00694 (14)	0.9178 (3)	0.0268 (6)	
H7	0.1336	0.0578	0.9433	0.032*	
C8	0.08665 (12)	−0.03538 (15)	0.9615 (3)	0.0312 (6)	
H8	0.0623	−0.0126	1.0152	0.037*	
C9	0.07908 (11)	−0.11011 (14)	0.9288 (3)	0.0299 (6)	
C10	0.11503 (12)	−0.14132 (14)	0.8479 (3)	0.0321 (6)	
H10	0.1106	−0.1923	0.8234	0.038*	
C11	0.15695 (11)	−0.09988 (13)	0.8024 (3)	0.0277 (6)	
H11	0.1808	−0.1226	0.7473	0.033*	
C12	0.03444 (13)	−0.15614 (17)	0.9807 (3)	0.0410 (7)	
H12A	0.0174	−0.1957	0.9055	0.061*	
H12B	0.0001	−0.1251	0.9875	0.061*	
H12C	0.0568	−0.1775	1.0827	0.061*	
C13	0.19299 (11)	0.20586 (13)	0.8382 (3)	0.0207 (5)	
H13	0.2355	0.2165	0.8521	0.025*	
C14	0.15332 (11)	0.26319 (13)	0.8662 (3)	0.0199 (5)	
C15	0.09753 (11)	0.24755 (14)	0.8943 (3)	0.0256 (5)	
H15	0.0839	0.1980	0.8929	0.031*	
C16	0.06239 (12)	0.30375 (14)	0.9239 (3)	0.0300 (6)	
H16	0.0247	0.2930	0.9434	0.036*	
C17	0.08210 (12)	0.37635 (14)	0.9251 (3)	0.0283 (6)	
H17	0.0574	0.4149	0.9448	0.034*	
C18	0.13694 (11)	0.39332 (13)	0.8982 (3)	0.0228 (5)	
H18	0.1501	0.4430	0.8997	0.027*	
C19	0.17279 (11)	0.33639 (13)	0.8688 (3)	0.0200 (5)	

### Atomic displacement parameters (Å^2^)

**Table:**

	*U*^11^	*U*^22^	*U*^33^	*U*^12^	*U*^13^	*U*^23^
S1	0.0284 (3)	0.0148 (3)	0.0310 (3)	−0.0006 (2)	0.0153 (3)	0.0012 (2)
O1	0.0262 (9)	0.0155 (9)	0.0319 (9)	0.0002 (7)	0.0153 (8)	0.0028 (7)
N1	0.0248 (11)	0.0157 (10)	0.0317 (11)	0.0003 (8)	0.0134 (9)	0.0030 (9)
N2	0.0256 (11)	0.0168 (10)	0.0300 (11)	−0.0006 (8)	0.0121 (9)	0.0008 (9)
N3	0.0205 (10)	0.0145 (10)	0.0235 (10)	0.0028 (8)	0.0093 (8)	−0.0001 (8)
N4	0.0222 (10)	0.0164 (10)	0.0252 (10)	0.0028 (8)	0.0108 (8)	0.0001 (8)
C1	0.0310 (15)	0.0409 (17)	0.0403 (16)	0.0033 (12)	0.0189 (13)	−0.0005 (13)
C2	0.0261 (13)	0.0246 (13)	0.0277 (13)	−0.0015 (10)	0.0108 (11)	−0.0009 (11)
C3	0.0277 (13)	0.0195 (13)	0.0274 (12)	−0.0009 (10)	0.0126 (11)	0.0021 (10)
C4	0.0210 (12)	0.0156 (12)	0.0218 (11)	−0.0009 (9)	0.0073 (10)	0.0002 (9)
C5	0.0219 (12)	0.0136 (11)	0.0229 (11)	0.0015 (9)	0.0070 (10)	0.0000 (9)
C6	0.0227 (12)	0.0188 (12)	0.0262 (12)	0.0002 (10)	0.0075 (10)	0.0069 (10)
C7	0.0330 (14)	0.0230 (13)	0.0274 (13)	0.0010 (11)	0.0140 (11)	0.0038 (11)
C8	0.0301 (14)	0.0366 (15)	0.0298 (13)	0.0002 (12)	0.0139 (11)	0.0059 (12)
C9	0.0250 (14)	0.0299 (15)	0.0322 (14)	−0.0059 (11)	0.0063 (11)	0.0107 (12)
C10	0.0265 (14)	0.0203 (13)	0.0462 (16)	−0.0027 (11)	0.0079 (12)	0.0056 (12)
C11	0.0250 (13)	0.0200 (13)	0.0377 (14)	0.0004 (10)	0.0099 (11)	0.0037 (11)
C12	0.0327 (16)	0.0453 (18)	0.0445 (17)	−0.0104 (13)	0.0125 (13)	0.0141 (14)
C13	0.0235 (12)	0.0190 (12)	0.0201 (11)	0.0002 (10)	0.0081 (10)	0.0014 (9)
C14	0.0238 (12)	0.0179 (12)	0.0186 (11)	0.0010 (9)	0.0079 (9)	0.0005 (9)
C15	0.0279 (13)	0.0201 (13)	0.0322 (13)	−0.0020 (10)	0.0145 (11)	−0.0018 (11)
C16	0.0297 (14)	0.0246 (14)	0.0428 (15)	−0.0012 (11)	0.0217 (12)	−0.0028 (12)
C17	0.0306 (14)	0.0240 (13)	0.0340 (14)	0.0053 (11)	0.0159 (12)	−0.0007 (11)
C18	0.0283 (13)	0.0169 (12)	0.0251 (12)	−0.0004 (10)	0.0115 (10)	0.0002 (10)
C19	0.0239 (12)	0.0199 (12)	0.0177 (11)	−0.0005 (10)	0.0088 (9)	0.0007 (10)

### Geometric parameters (Å, °)

**Table:**

S1—C4	1.744 (2)	C7—H7	0.9500
S1—C3	1.812 (2)	C8—C9	1.389 (4)
O1—C19	1.357 (3)	C8—H8	0.9500
O1—H1	0.95 (3)	C9—C10	1.394 (4)
N1—C4	1.310 (3)	C9—C12	1.508 (3)
N1—N2	1.388 (3)	C10—C11	1.382 (3)
N2—C5	1.318 (3)	C10—H10	0.9500
N3—C5	1.377 (3)	C11—H11	0.9500
N3—C4	1.386 (3)	C12—H12A	0.9800
N3—N4	1.407 (3)	C12—H12B	0.9800
N4—C13	1.287 (3)	C12—H12C	0.9800
C1—C2	1.529 (3)	C13—C14	1.455 (3)
C1—H1A	0.9800	C13—H13	0.9500
C1—H1B	0.9800	C14—C19	1.399 (3)
C1—H1C	0.9800	C14—C15	1.403 (3)
C2—C3	1.523 (3)	C15—C16	1.377 (3)
C2—H2A	0.9900	C15—H15	0.9500
C2—H2B	0.9900	C16—C17	1.392 (3)
C3—H3A	0.9900	C16—H16	0.9500
C3—H3B	0.9900	C17—C18	1.382 (3)
C5—C6	1.470 (3)	C17—H17	0.9500
C6—C7	1.388 (3)	C18—C19	1.397 (3)
C6—C11	1.401 (3)	C18—H18	0.9500
C7—C8	1.390 (3)		
			
C4—S1—C3	98.71 (11)	C9—C8—H8	119.1
C19—O1—H1	114 (2)	C7—C8—H8	119.1
C4—N1—N2	107.00 (19)	C8—C9—C10	117.4 (2)
C5—N2—N1	109.10 (19)	C8—C9—C12	121.5 (3)
C5—N3—C4	105.69 (18)	C10—C9—C12	121.1 (2)
C5—N3—N4	123.09 (18)	C11—C10—C9	121.6 (2)
C4—N3—N4	130.37 (19)	C11—C10—H10	119.2
C13—N4—N3	114.81 (19)	C9—C10—H10	119.2
C2—C1—H1A	109.5	C10—C11—C6	120.5 (2)
C2—C1—H1B	109.5	C10—C11—H11	119.8
H1A—C1—H1B	109.5	C6—C11—H11	119.8
C2—C1—H1C	109.5	C9—C12—H12A	109.5
H1A—C1—H1C	109.5	C9—C12—H12B	109.5
H1B—C1—H1C	109.5	H12A—C12—H12B	109.5
C3—C2—C1	110.6 (2)	C9—C12—H12C	109.5
C3—C2—H2A	109.5	H12A—C12—H12C	109.5
C1—C2—H2A	109.5	H12B—C12—H12C	109.5
C3—C2—H2B	109.5	N4—C13—C14	121.1 (2)
C1—C2—H2B	109.5	N4—C13—H13	119.4
H2A—C2—H2B	108.1	C14—C13—H13	119.4
C2—C3—S1	114.79 (17)	C19—C14—C15	119.3 (2)
C2—C3—H3A	108.6	C19—C14—C13	118.3 (2)
S1—C3—H3A	108.6	C15—C14—C13	122.5 (2)
C2—C3—H3B	108.6	C16—C15—C14	120.2 (2)
S1—C3—H3B	108.6	C16—C15—H15	119.9
H3A—C3—H3B	107.5	C14—C15—H15	119.9
N1—C4—N3	109.80 (19)	C15—C16—C17	119.9 (2)
N1—C4—S1	124.89 (18)	C15—C16—H16	120.0
N3—C4—S1	125.18 (17)	C17—C16—H16	120.0
N2—C5—N3	108.4 (2)	C18—C17—C16	121.1 (2)
N2—C5—C6	124.0 (2)	C18—C17—H17	119.5
N3—C5—C6	127.6 (2)	C16—C17—H17	119.5
C7—C6—C11	118.4 (2)	C17—C18—C19	119.1 (2)
C7—C6—C5	123.9 (2)	C17—C18—H18	120.4
C11—C6—C5	117.7 (2)	C19—C18—H18	120.4
C6—C7—C8	120.4 (2)	O1—C19—C18	122.4 (2)
C6—C7—H7	119.8	O1—C19—C14	117.2 (2)
C8—C7—H7	119.8	C18—C19—C14	120.4 (2)
C9—C8—C7	121.8 (2)		
			
C4—N1—N2—C5	0.2 (3)	C5—C6—C7—C8	−179.9 (2)
C5—N3—N4—C13	154.6 (2)	C6—C7—C8—C9	1.0 (4)
C4—N3—N4—C13	−37.5 (3)	C7—C8—C9—C10	−0.9 (4)
C1—C2—C3—S1	176.26 (18)	C7—C8—C9—C12	178.2 (2)
C4—S1—C3—C2	−80.05 (19)	C8—C9—C10—C11	0.4 (4)
N2—N1—C4—N3	1.0 (3)	C12—C9—C10—C11	−178.8 (2)
N2—N1—C4—S1	−175.07 (16)	C9—C10—C11—C6	0.0 (4)
C5—N3—C4—N1	−1.8 (2)	C7—C6—C11—C10	0.0 (4)
N4—N3—C4—N1	−171.2 (2)	C5—C6—C11—C10	179.4 (2)
C5—N3—C4—S1	174.29 (17)	N3—N4—C13—C14	−179.28 (19)
N4—N3—C4—S1	4.8 (3)	N4—C13—C14—C19	−161.9 (2)
C3—S1—C4—N1	11.7 (2)	N4—C13—C14—C15	19.7 (3)
C3—S1—C4—N3	−163.77 (19)	C19—C14—C15—C16	0.1 (4)
N1—N2—C5—N3	−1.3 (3)	C13—C14—C15—C16	178.4 (2)
N1—N2—C5—C6	177.6 (2)	C14—C15—C16—C17	0.3 (4)
C4—N3—C5—N2	1.9 (2)	C15—C16—C17—C18	−0.5 (4)
N4—N3—C5—N2	172.28 (19)	C16—C17—C18—C19	0.3 (4)
C4—N3—C5—C6	−177.0 (2)	C17—C18—C19—O1	−179.8 (2)
N4—N3—C5—C6	−6.6 (4)	C17—C18—C19—C14	0.2 (3)
N2—C5—C6—C7	164.0 (2)	C15—C14—C19—O1	179.7 (2)
N3—C5—C6—C7	−17.3 (4)	C13—C14—C19—O1	1.2 (3)
N2—C5—C6—C11	−15.3 (3)	C15—C14—C19—C18	−0.3 (3)
N3—C5—C6—C11	163.4 (2)	C13—C14—C19—C18	−178.7 (2)
C11—C6—C7—C8	−0.6 (4)		

### Hydrogen-bond geometry (Å, °)

**Table:**

*D*—H···*A*	*D*—H	H···*A*	*D*···*A*	*D*—H···*A*
O1—H1···N1^i^	0.95 (3)	2.58 (3)	3.464 (3)	155 (3)
O1—H1···N2^i^	0.95 (3)	1.71 (3)	2.658 (2)	175 (3)

Symmetry codes: (i) −*x*+1/2, *y*+1/2, −*z*+3/2.
